# Isolation of Endophytic Bacteria from Kentucky Bluegrass and the Biocontrol Effects of *Neobacillus* sp. 718 on Powdery Mildew

**DOI:** 10.3390/plants14243758

**Published:** 2025-12-10

**Authors:** Yinping Liang, Fan Wu, Yining Zhang, Zhanchao Guo, Lingjuan Han, Peng Gao, Xiang Zhao, Huisen Zhu

**Affiliations:** 1 College of Grassland Science, Shanxi Agricultural University, Jinzhong 030801, China; z20223228@stu.sxau.edu.cn (F.W.); 20233294@stu.sxau.edu.cn (Y.Z.); 202430308@stu.sxau.edu.cn (Z.G.); hanlj@sxau.edu.cn (L.H.); gaopeng@sxau.edu.cn (P.G.); sxndzhaox@sxau.edu.cn (X.Z.); 2 Bikqi Town Government, Hohhot 010107, China

**Keywords:** antagonistic activity, endophytes, fungal disease, microbial community diversity, turfgrass

## Abstract

Kentucky bluegrass powdery mildew, caused by the fungus *Blumeria graminis* f. sp. *poae*, is a destructive disease affecting *Poa pratensis* L. In this study, endophytic bacteria were isolated from the resistant Kentucky bluegrass cultivar ‘Taihang’. Employing a combination of conidia germination inhibition assays and control efficacy tests, the biocontrol endophytic bacterial strains were screened. The impact of inoculation with the powdery mildew pathogen and biocontrol endophytic bacteria on the difference in endophytic bacterial community in the leaves of Kentucky bluegrass were studied via Illumina Miseq high-throughput 16S ribosomal RNA gene sequencing technology. A total of 18 endophytic bacterial isolates were obtained from ‘Taihang’, belonging to 3 phyla: Proteobacteria (3 isolates), Actinobacteria (6 isolates), and Firmicutes (9 isolates). The conidia germination assay revealed that isolates 6213 (*Bacillus* sp.) and 718 (*Neobacillus* sp.) exhibited the strongest inhibitory against *Blumeria graminis* f. sp. *poae*, with inhibition rate exceeding 80%. Isolate 718 exhibited superior control efficacy over strain 6213. A concentration of 10^9^ colony-forming units per milliliter (CFU/mL) was the most effective in suppressing powdery mildew on Kentucky bluegrass. The abundance of Proteobacteria on Kentucky bluegrass after the application of isolate 718 may enhance the resistance of Kentucky bluegrass to powdery mildew, and the dominant endophytic bacterial communities were Burkholderiales, Burkholderiaceae and *Cupriavidus*, indicating that the application of isolate 718 modulated the plant’s response to powdery mildew infection. These results demonstrate that isolate 718 enhanced the resistance of Kentucky bluegrass against powdery mildew by reshaping the endophytic bacterial community within the leaves. These findings provide molecular insights into plant−pathogen−endophytic bacteria interactions and support the development of sustainable strategies, eco-friendly strategies for plant diseases management.

## 1. Introduction

Kentucky bluegrass (*Poa pratensis* L.) is a perennial cold-season grass widely utilized as a turfgrass and valuable forage due to its strong ecological adaptability and asexual reproduction via tiller nodes and rhizomes [1,2,3]. Biologically, it is characterized by a dense sod-forming growth habit, early spring green-up, and considerable genetic diversity, which supports its persistence across a range of temperate environments [4]. However, its widespread cultivation is limited by susceptibility to diseases and pests, as well as sensitivity to heat, drought and salinity [1,5,6]. In Shanxi Province, Kentucky bluegrass is valued for rapid establishment, grazing tolerance, and utility in urban and rural landscaping, pasture and ecological restoration. However, Kentucky bluegrass is frequently challenged by powdery mildew.

Powdery mildew, caused by *Blumeria graminis* DC. Speer, is a common stem and leaf disease in turfgrass [7,8]. It frequently occurs in shady area with high relative humidity or in the seed production field [7,9]. This disease can infect a variety of grass species, such as *Agrostis stolonifera*, *Cynodon dactylon* and *Poa pratensis*. As a polycyclic disease, powdery mildew can spread by wind, air currents or insects, leading to a large−scale epidemic. These outbreaks severely reduce the turfgrass ornamental and forage value and complicate disease management efforts [10,11]. In Kentucky bluegrass, this disease is caused by the forma specialis *B. graminis* f. sp. *poae* [12]. The disease affects above-ground organs of Kentucky bluegrass, impairing key physiological processes such as photosynthesis, stomatal conductance, and transpiration rate, thereby significantly compromising plant health and productivity [12].

Breeding resistant cultivars is a key strategy to combat powdery mildew. However, the high phylogenetic diversity, strong adaptability, and rapid mutation rate of the pathogen often lead to the breakdown of plant resistance. In practice, chemical control remains a primary approach for managing powdery mildew, such as Triadimefon powder [13]. Nevertheless, excessive reliance on chemical fungicides would induce resistance in pathogens and cause environmental pollution. As a result, biological control agents are being increasingly adopted. There is a growing trend to replace traditional chemical methods with sustainable biocontrol strategies [14].

Endophytic bacteria colonize the inside of plant tissues and play a crucial role in enhancing plant resistance to biotic stress [15]. They contribute to plant defense through the production of antagonistic compounds and the induced systemic resistance [16]. Plants possess inherent physical and chemical barriers that recognize pathogens and activate signal transduction pathways to mount defense responses. Endophytic bacteria elicit both direct and indirect mechanisms of disease resistance in plants [17,18,19]. For example, *Bacillus* species synthesize lipopeptide antibiotics and volatile organic compounds [20,21], while *Streptomyces* species produce compounds such as streptomycin and tetracycline [22]. In addition, *Bacillus* can also enhance the defense ability of plants by inducing systemic resistance. For example, *Bacillus* subtilis can enhance the resistance of plants to pathogens by activating jasmonic acid (JA) and salicylic acid (SA) signaling pathways [23]. Additionally, endophytic bacteria enhance plant tolerance to abiotic stresses such as drought through molecular and biochemical modifications [24,25,26]. Endophytic *Stenotrophomonas* SaRB5 inoculation enhanced willow growth and Cd phytoremediation efficiency by regulating hormones, promoting Cd translocation, and facilitating Cd accumulation in the less metabolically active zones [27]. Similarly, under salt stress, endophytic bacteria help maintain cellular function by stimulating the synthesis of antioxidant enzymes to scavenge reactive oxygen species. Inoculation with the endophytic bacterium *Bacillus cereus* KP120 increased proline content and antioxidant enzyme activity in *Arabidopsis thaliana*, thereby enhancing salt stress tolerance [28]. In summary, endophytic bacteria significantly bolster plant resilience to disease and environmental stresses by activating various biochemical response pathways within the host [29].

Plant endophytes play a significant role in the biological control of plant diseases. Sustainable agriculture emphasizes the need for eco-friendly biocontrol strategies [30]. Unlike chemical pesticides, which can lead to pathogen resistance and pose serious risks to human health, animals, and the environment, plant endophytes offer a promising alternative. They not only effectively inhibit pathogens but also are non-toxic, environmentally benign, and less likely to induce resistance [31]. Compared to other microorganisms, endophytic bacteria exhibit distinct advantages in the field of biocontrol. As natural inhabitants of plant tissues, they are widely distributed within their hosts. This internal colonization allows them to utilize the abundant nutrients provided by the plant while being shielded from external stressors such as ultraviolet radiation and extreme weather fluctuations. Such a stable and privileged microecological niche enhances their potential for biocontrol, enabling endophytic bacteria to persistently exert beneficial effects within the host [32]. While their role in biocontrol is increasingly recognized, little is known about their function in the Kentucky bluegrass–powdery mildew pathosystem. In particular, the potential of endophytes in preventing *B. graminis* and their influence on the plant’s microbiome under disease pressure remain largely unexplored.

Based on the existing knowledge and the rationale of our study, we hypothesized that the resistant Kentucky bluegrass variety ‘Taihang’ harbors specific endophytic bacteria with potent biocontrol activity against *B. graminis* f. sp. *poae*. We further hypothesized that a biocontrol isolate reshapes in the host’s endophytic bacterial community and this shift plays a key role in strengthening the plant defense against powdery mildew infection. In this study, endophytic bacteria were isolated from the leaf and root samples of the resistant Kentucky bluegrass variety ‘Taihang’. Among the endophytic bacteria isolated, the isolate *Neobacillus* sp. 718 with the best function in inhibiting conidia germination was expected to be a valuable microbial resource for the biocontrol of powdery mildew. Furthermore, the leaf endophytic bacteria communities of Kentucky bluegrass inoculated with *Neobacillus* sp. 718 and powdery mildew pathogen *B. graminis* f. sp. *poae* were investigated via high-throughput 16S rRNA gene sequencing technology. The findings of this study will provide new insights into the application of endophytic bacteria for the biological control of powdery mildew and the associated dynamics of the plant endophytic bacteria.

## 2. Results

### 2.1. Molecular Biological Identification and Classification of Endophytic Bacteria Isolated from Kentucky Bluegrass

**Figure 1:**
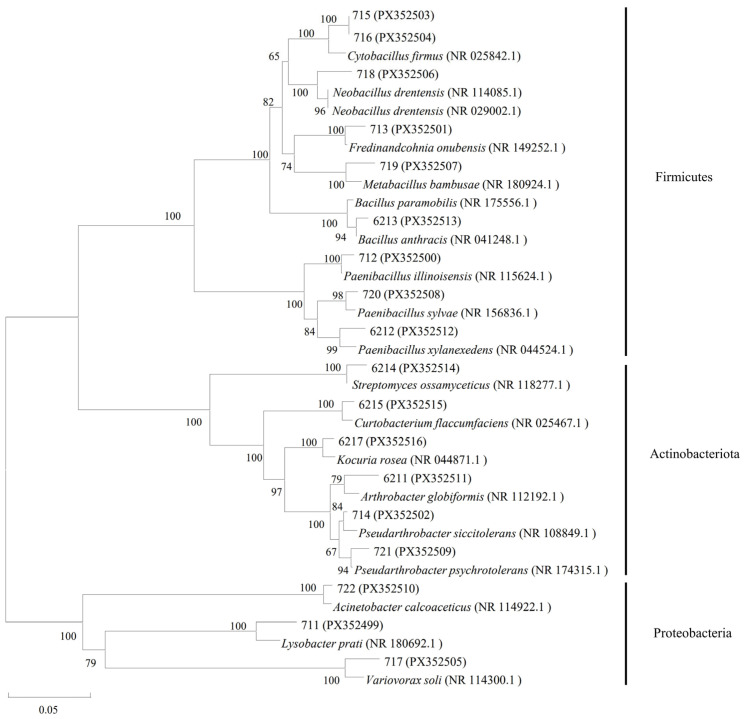
Phylogenetic tree of endophytic bacteria isolated from *Poa pratensis* L. was constructed based on 16S rRNA gene sequences. The serial numbers in parentheses are GenBank accession numbers; The numbers on the nodes indicate the support percentages of bootstrap. The scale 0.05 represents 5% nucleotide difference.

A total of 7 endophytic bacteria were isolated from the roots of Kentucky bluegrass (isolates 711, 712, 713, 714, 715, 716, 717), and 11 endophytic bacteria were isolated from the leaves of Kentucky bluegrass (isolates 718, 719, 720, 721, 722, 6211, 6212, 6213, 6214, 6215, 6217). The 16S rRNA gene sequences of the 18 isolates were submitted to the NCBI GenBank and accession numbers were obtained and are shown in Table 1. Phylogenetic analysis classified these isolates into three phyla: Proteobacteria (3 isolates), Actinobacteria (6 isolates), and Firmicutes (9 isolates) (Figure 1). The Proteobacteria phylum comprises isolates 711 (*Lysobacter* sp.), 717 (*Variovorax* sp.), and 722 (*Acinetobacter* sp.). The Actinobacteria phylum comprises isolates 6211 (*Arthrobacter* sp.), 6214 (*Streptomyces* sp.), 6215 (*Curtobacterium* sp.), 6217 (*Kocuria* sp.), 714 (*Pseudarthrobacter* sp.), and 721 (*Pseudarthrobacter* sp.). The Firmicutes phylum comprises isolates 6212 (*Paenibacillus* sp.), 6213 (*Bacillus* sp.), 712 (*Paenibacillus* sp.), 713 (*Fredinandcohnia* sp.), 715 (*Cytobacillus* sp.), 716 (*Cytobacillus* sp.), 718 (*Neobacillus* sp.), 719 (*Metabacillus* sp.), and 720 (*Paenibacillus* sp.).

### 2.2. The Influence of Endophytic Bacteria Isolated from Kentucky Bluegrass on the Germination Inhibition of Powdery Mildew Conidia

**Figure 2:**
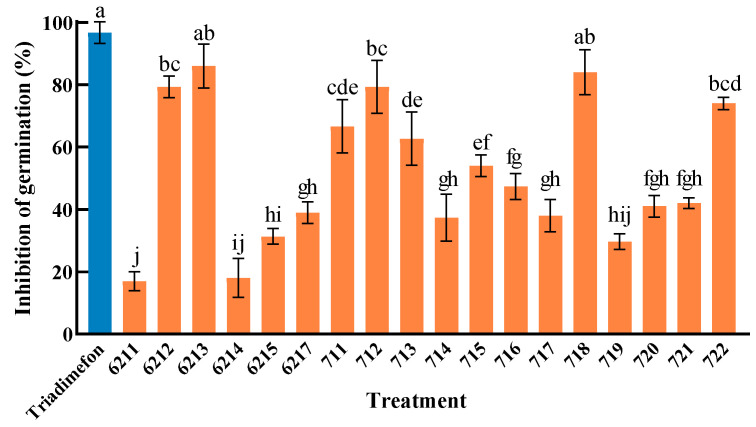
Inhibition rate of endophytic bacteria of *Poa pratensis* L. on conidia germination of powdery mildew. The experiment was conducted with three biological replicates. Each replicate consisted of 50 conidia of *B. graminis* f.sp. *poae*. The error bars represent the standard deviation (SD). The data are presented in the form of the mean ± SD. Different lowercase letters indicate significant differences between different treatments (*p* < 0.05).

Through the determination of the inhibitory effects of different endophytic bacterial isolates in Kentucky bluegrass on the germination of powdery mildew conidia, a preliminary screening was conducted. The results showed that all 18 endophytic bacterial isolates exhibited certain inhibitory effects on the germination of powdery mildew conidia, with the germination inhibition rates ranging from 17% to 97% (Figure 2). Statistical analysis of the conidia germination inhibition rates clearly distinguished the tested endophytic bacterial isolates into three efficacy groups (Figure 2). Isolates 6213 and 718 formed a high-efficacy group, the inhibition rates of which were not statistically different from that of the chemical fungicide 20% triadimefon EC solution. A middle-efficacy group, including isolates 6212, 712, and 722, also demonstrated strong suppression without significant with that of isolates 6213 and 718. The remaining isolates constituted a low-efficacy group with moderate significant activity. This clear stratification highlights isolates 6213 and 718 as the most promising candidates for further biocontrol development.

### 2.3. The Biological Control Effect of Isolate 6213 and Isolate 718 on Kentucky Bluegrass Powdery Mildew

**Figure 3:**
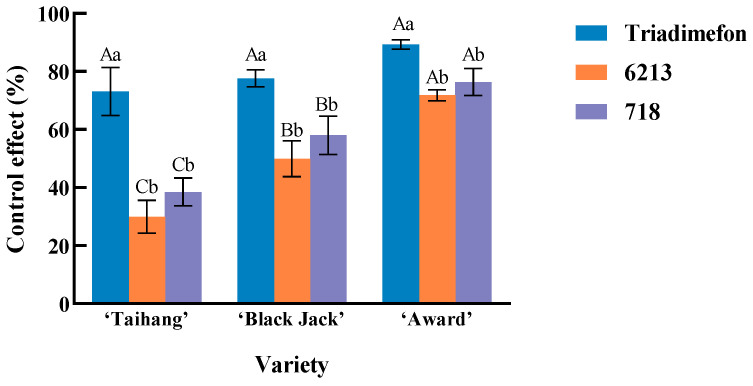
Effects of different treatments on the control effect of powdery mildew of three varieties of *Poa pratensis* L. Different lowercase letters indicate significant differences between different treatments of the same variety (*p* < 0.05). Capital letters indicate significant differences between different varieties of the same treatment (*p* < 0.05).

The biological control efficacy of endophytic bacteria against powdery mildew was calculated as the percentage reduction in the disease index relative to the control. The control effects of isolates 718 and 6213 against powdery mildew in Kentucky bluegrass were significantly lower than that of 20% triadimefon EC (*p* < 0.05) across three varieties: the moderately resistant ‘Taihang’, the moderately susceptible ‘Black Jack’, and the highly susceptible ‘Award’. Although isolate 718 consistently outperformed isolate 6213 in all varieties, the difference between the two isolates was not statistically significant. After treatment with 20% triadimefon EC, there was no significant difference observed among the disease control effect of the three varieties. In contrast, when treated with either isolate 718 or 6213, ‘Award’ showed a significantly higher control effect than ‘Black Jack’ (*p* < 0.05), which in turn was significantly higher than that of ‘Taihang’ (*p* < 0.05) (Figure 3). These results indicate that the control efficacy of endophytes is closely linked to the plant host’s resistance level. This may be because the immune systems of susceptible varieties may be more activated, which makes them respond more strongly to beneficial endophytes that can activate plant system resistance. For field application, this variety-specificity suggests biocontrol endophytes should be used preferentially in susceptible varieties to maximize ecological and economic value.

### 2.4. The Biological Control Effect of Different Concentrations of Isolate 718 on Kentucky Bluegrass Powdery Mildew

**Figure 4:**
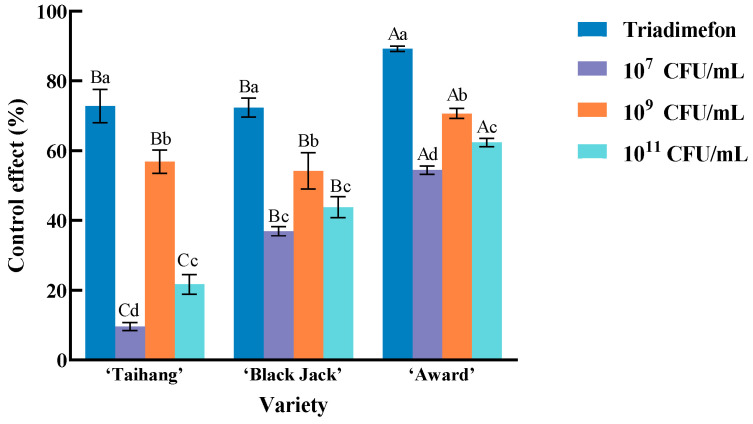
Effects of different concentration treatments on the control effect of powdery mildew of three varieties of *Poa pratensis* L. Different lowercase letters indicate significant differences between different treatments of the same variety (*p* < 0.05). Capital letters indicate significant differences between different varieties of the same treatment (*p* < 0.05).

The varieties ‘Taihang’, ‘Black Jack’, and ‘Award’ were sprayed with 20% triadimefon EC or bacterial suspension solutions of isolate 718 at concentrations of 10^7^, 10^9^, and 10^11^ CFU/mL, respectively. The control efficacy against powdery mildew in Kentucky bluegrass was then evaluated. The results indicated that the control effect of 20% triadimefon EC was significantly higher than that of the 10^9^ CFU/mL treatment (*p* < 0.05), which in turn was significantly higher than the 10^11^ CFU/mL treatment (*p* < 0.05), and the 10^11^ CFU/mL treatment was superior to the 10^7^ CFU/mL treatment. Under 20% triadimefon EC treatment, ‘Award’ showed a significantly higher control effect than both ‘Taihang’ and ‘Black Jack’ (*p* < 0.05), with no significant difference between the latter two. At 10^7^ or 10^11^ CFU/mL, the control effect in ‘Award’ was significantly higher than in ‘Black Jack’, which in turn was significantly higher than in ‘Taihang’ (*p* < 0.05). At 10^9^ CFU/mL, no significant difference was observed between ‘Taihang’ and ‘Black Jack’, but both were significantly lower than ‘Award’ (*p* < 0.05). Overall, the 10^9^ CFU/mL suspension of isolate 718 demonstrated the best control efficacy against powdery mildew among the bacterial treatments. Therefore, this concentration was selected as optimal for subsequent experiments (Figure 4).

### 2.5. Operational Taxonomic Units (OTU) Statistics and Analysis

**Figure 5:**
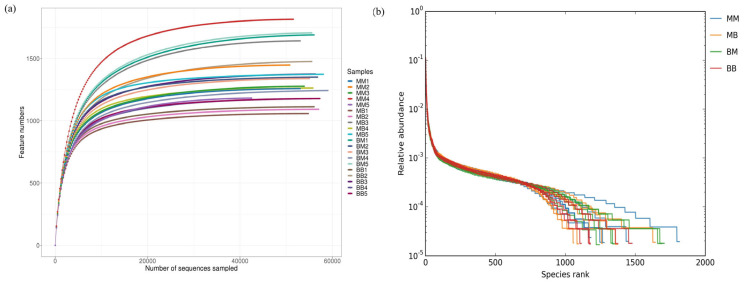
The bacterial dilution curve (**a**) and bacterial rank−abundance curve (**b**) of different *Poa pratensis* L. treatments. The sample groups included MM (application of water without inoculation of powdery mildew), MB (application of water with inoculation of powdery mildew), BM (application of isolate 718 bacterial suspension without inoculation of powdery mildew), BB (application of isolate 718 bacterial suspension with inoculation of powdery mildew).

The results show that the bacterial dilution curves of Kentucky bluegrass after different treatments exhibited a rapid initial rise followed by a gradual plateau (Figure 5a). The leveling-off of the curve suggests that the sequencing depth was adequate and reasonable, as further increases in sequencing volume would not lead to a significant rise in species richness. The rank–abundance curves were used to assess species richness and evenness in each Kentucky bluegrass sample (Figure 5b). A flatter curve with a wider span reflects higher species evenness and greater richness in the community.

**Figure 6:**
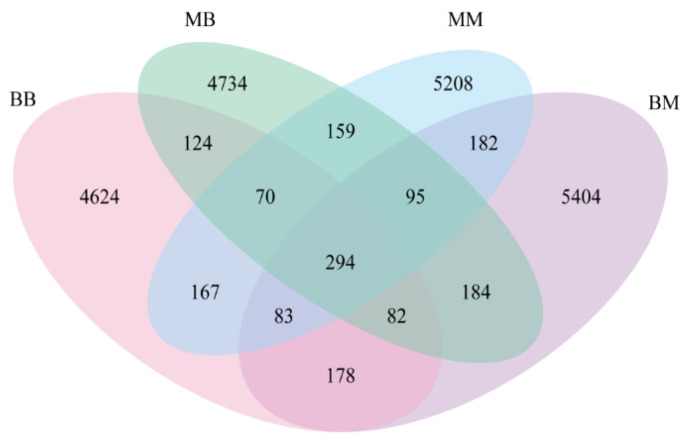
The OTUs distribution of bacterial community in different *Poa pratensis* L. treatments. The sample groups included MM (application of water without inoculation of powdery mildew), MB (application of water with inoculation of powdery mildew), BM (application of isolate 718 bacterial suspension without inoculation of powdery mildew), BB (application of isolate 718 bacterial suspension with inoculation of powdery mildew).

The Venn diagram illustrating bacterial community OTUs across different treatments of Kentucky bluegrass is shown in Figure 6. The BM treatment group contained the highest number of OTUs (6502) that was 3.9% higher than that in the MM treatment group, potentially linked to its response to powdery mildew infection following application of isolate 718, The number of OTUs in the MB and BB treatment groups was 8.25% and 10.16% lower, respectively, compared to the MM treatment group, which indicated powdery mildew has a negative impact on the bacterial community. A core set of 294 OTUs was shared among all four treatments, which implied a stable microbial component unaffected by the treatment. Each group also possessed a high proportion of unique OTUs that are of considerable importance for understanding the response of Kentucky bluegrass to powdery mildew infection following treatment with isolate 718.

### 2.6. Analysis of Bacterial Community Diversity

**Figure 7:**
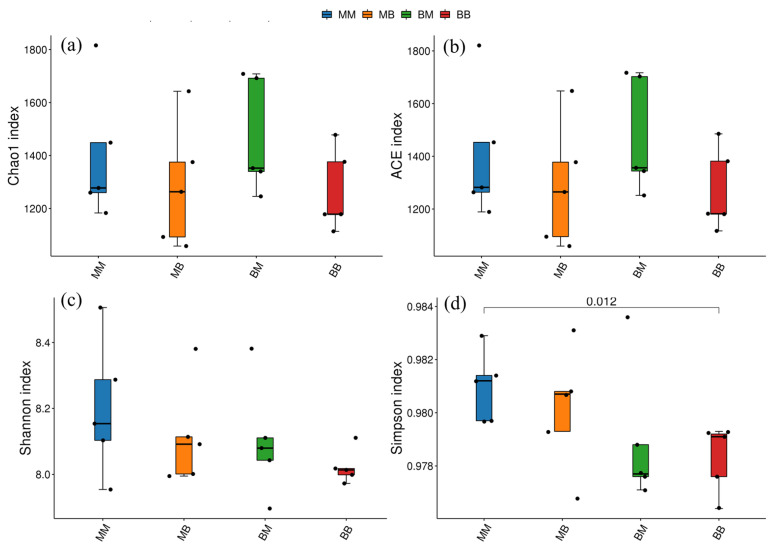
Analysis of α diversity of endophytic bacterial community in *Poa pratensis* L. after different treatments ((**a**): Chao1 index diagram; (**b**): ACE index diagram; (**c**): Shannon index diagram; (**d**): Simpson index diagram). The sample groups included MM (application of water without inoculation of powdery mildew), MB (application of water with inoculation of powdery mildew), BM (application of isolate 718 bacterial suspension without inoculation of powdery mildew), BB (application of isolate 718 bacterial suspension with inoculation of powdery mildew).

The alpha diversity of the endophytic bacterial community in *Poa pratensis* L. was evaluated based on four indices: Chao1 and Ace (reflecting species richness), and Shannon and Simpson (reflecting species diversity). As shown in Figure 7, the richness estimates ranked the groups as BM > MM > MB > BB, but no significant differences were observed. Endophytic bacteria 718 may have increased the richness of the endophytic bacterial community, thereby enhancing the disease resistance of plants. Conversely, a significant difference was observed in the Simpson index, with the MM group showing significantly higher diversity than the BB group. This decline suggests that the beneficial isolates successfully dominated the microbial niche, potentially through competitive exclusion or the production of antimicrobial compounds. The absence of significance in other indices suggests that this variation was driven by shifts in species evenness rather than richness.

### 2.7. Taxonomy Richness Analysis

**Figure 8:**
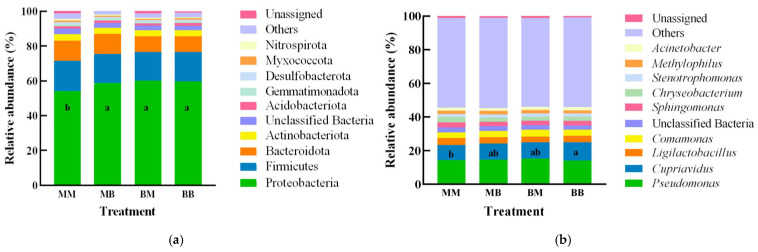
Species distribution map of the bacterial endophytes in *Poa pratensis* L. after different treatments at phylum (**a**) and genus (**b**) levels. The sample groups included MM (application of water without inoculation of powdery mildew), MB (application of water with inoculation of powdery mildew), BM (application of isolate 718 bacterial suspension without inoculation of powdery mildew), BB (application of isolate 718 bacterial suspension with inoculation of powdery mildew). Different lowercase letters indicate significant differences between different treatments (*p* < 0.05).

As shown in Figure 8a, which displays the relative abundance of the top 10 bacterial phyla and genera across different treatments of *Poa pratensis* L., the bacterial community was dominated by the phylum *Proteobacteria*, followed by *Firmicutes*, *Bacteroidota*, *Actinobacteria*, *Acidobacteriota*, *Gemmatimonadota*, *Desulfobacterota*, *Myxococcota*, and *Nitrospirota*. At the phylum level, comparative analysis revealed the following differences: The abundance of *Proteobacteria* in the MM treatment was distinctly lower, showing a significant difference from the BB, BM, and MB treatments (*p* < 0.05), among which there were no significant differences. The isolate 718 might help maintain a microbiome structure richer in beneficial bacteria Proteobacteria, which could contribute to the observed disease suppression by enhancing competition for ecological niches or by direct antagonism against the pathogen. The abundance of remaining phyla among BB, BM, MB and MM showed no significant differences.

At the genus level, *Pseudomonas* was the most abundant genus, followed by *Cupriavidus*, *Ligilactobacillus*, *Comamonas*, *Sphingomonas*, *Chryseobacterium*, *Stenotrophomonas*, *Methylophilus*, and *Acinetobacter* (Figure 8b). The abundance of *Cupriavidus* in the BB treatment was significantly higher than that in MM treatment (*p* < 0.05). The abundance of remaining genera among BB, BM, MB and MM showed no significant differences. The marked enrichment of *Cupriavidus* in the BB treatment group holds significant ecological implications for disease suppression.

### 2.8. Analysis of Differences Between Groups

**Figure 9:**
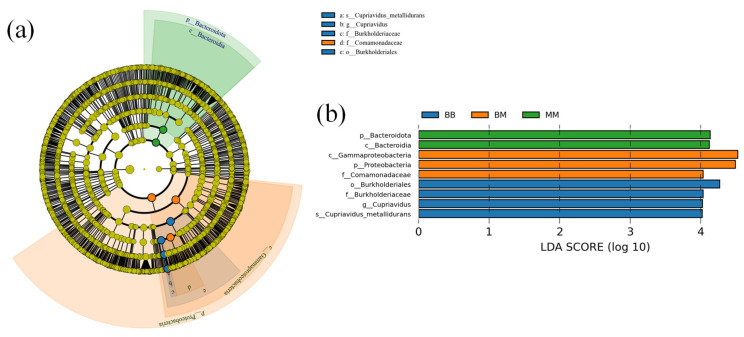
LEfSe analysis evolutionary branch diagram (**a**) and LDA value distribution histogram (**b**). Circles radiating from the inside to the outside of Figure (**a**) represent taxonomic levels from phylum to species; each small circle at a different taxonomic level represents a taxa at that level, and the size of the diameter of the circle is proportional to the size of the relative abundance. The sample groups included MM (application of water without inoculation of powdery mildew), BM (application of isolate 718 bacterial suspension without inoculation of powdery mildew), BB (application of isolate 718 bacterial suspension with inoculation of powdery mildew).

LEfSe (Line Discriminant Analysis (LDA) Effect Size) analysis was employed to distinguish taxa with statistically significant differences in abundance among different *Poa pratensis* L. samples. As shown in Figure 9, there were two dominant endophytic bacterial communities in the MM group of *Poa pratensis* L., namely Bacteroidota and Bacteroidia; three dominant endophytic bacterial communities in the BM group of *Poa pratensis* L., namely Proteobacteria, Gammaproteobacteria and Comamonadaceae; four dominant endophytic bacterial communities in the BB group of *Poa pratensis* L., namely Burkholderiales, Burkholderiaceae, *Cupriavidus* and *Cupriavidus metallidurans*, while no dominant endophytic bacterial communities with significant abundance differences were found in the MB group of *Poa pratensis* L. Critically, the consortium of biomarkers enriched in the BB group (Burkholderiales, Burkholderiaceae, *Cupriavidus*) provides strong clues regarding potential biocontrol mechanisms.

## 3. Discussion

### 3.1. Endophytic Bacteria in Kentucky Bluegrass

Endophytic bacteria have demonstrated significant research value and broad application prospects in promoting sustainable green agriculture, ecological environment protection and restoration, as well as plant disease prevention and control. While numerous studies have reported the isolation and utilization of endophytic bacteria for controlling plant diseases, research focusing on endophytic bacteria in Kentucky bluegrass remains limited. In this study, 18 endophytic bacterial isolates were successfully isolated from resistant varieties of Kentucky bluegrass. This number is comparable to those reported in previous studies—such as 38 isolates from *Tinospora cordifolia* leaf and stem tissues [33] and 19 endophytic bacteria isolates from *Theobroma cacao* plants in Brazil [34]—supporting the feasibility and reliability of the isolation methodology employed here. These 18 endophytic bacterial isolates hold considerable research significance. As integral components of the plant micro-ecosystem, endophytic bacteria play crucial roles in enhancing plant growth, disease resistance, and ecological adaptability. Endophytic *Pantoea* sp. EEL5 with multiple plant growth-promoting traits and abiotic stress resistance was isolated from *Elytrigia elongate* leaves [35]. Endophytic bacterial strain H13, was isolated from rapeseed and was demonstrated to possess biocontrol activity against clubroot [36]. Thus, the isolates obtained in this study provide valuable resources for further elucidating the mechanisms by which endophytic bacteria contribute to the biocontrol of powdery mildew in Kentucky bluegrass.

Molecular identification and phylogenetic analysis classified the 18 endophytic isolates at the phylum level into three groups: 3 isolates of Proteobacteria, 6 of Actinobacteria, and 9 of Firmicutes. These findings align with existing literature indicating that endophytic bacterial communities in plants are predominantly composed of Firmicutes, Bacteroidetes, Proteobacteria, and Actinobacteria [37]. Among them, seven isolates were isolated from the roots, and the remaining 11 isolates were isolated from the leaves. The differences between these two groups may stem from their differential capacity to colonize specific plant niches [38,39]. Notably, genus such as *Bacillus* is frequently reported as plant growth-promoting and biocontrol agents in a variety of host plants. Their presence in Kentucky bluegrass suggests a conserved functional role in maintaining plant health. To assess whether these isolates represent novel taxa, it is noteworthy that the genus *Fredinandcohnia* (isolate 713) is reported here for the first time as plant endophyte. The specific isolate isolated from resistant Kentucky bluegrass may thus possess novel functional attributes.

### 3.2. Biocontrol Potential of Endophytic Bacteria Against Kentucky Bluegrass Powdery Mildew

At the genus level, the isolates included representatives of *Neobacillus*, *Bacillus* and *Acinetobacter* with the better function of inhibiting spore germination. *Paenibacillus* is is a prominent genus among plant endophytes, with numerous studies highlighting its potential in promoting plant growth and controlling diseases. Several species within this genus have been documented to possess biocontrol properties, including *P. polymyxa*, *P. alvei*, *P. brasilensis*, *P. dendritiformis*, *P. ehimensis*, *P. elgii*, *P. kobensis*, *P. lentimorbus*, *P. macerans*, *P. peoriae*, and *P. thiaminolyticus* [40]. In this study, *Paenibacillus* sp. 6212 exhibited strong inhibition against powdery mildew conidia, with an inhibition rate of 79%, further confirming the value of this genus in plant disease biocontrol.

*Bacillus* represents one of the most extensively studied and predominant genera of plant endophytes [41]. It can promote plant growth and control plant pathogens through multiple mechanisms, such as by improving nutrient availability, modulating phytohormone homeostasis, and producing antimicrobials [42]. In the present work, *Bacillus* sp. isolate 6213 showed excellent suppression of powdery mildew conidial germination, with an inhibition rate of 86%, and demonstrated high control efficacy against Kentucky bluegrass powdery mildew, second only to that of isolate 718. *Neobacillus* is a genus that has been established relatively recently. Although its functions in plant systems remain underexplored, its potential in synthesizing bioactive compounds and facilitating biocatalysis has attracted growing interest. For example, Routhu et al. reported the isolation of antifungal cyclic peptides from *N. drentensis*, suggesting its potential use in biocontrol [43]. Similarly, Dawei et al. demonstrated that inulosucrase from *N. bataviensis* can efficiently catalyze the conversion of sucrose into low molecular weight inulin, which holds significance for the food industry and functional food development [44]. In this study, isolate 718 displayed the highest control efficacy against Kentucky bluegrass powdery mildew in both conidial germination inhibition assays and indoor efficacy trials, providing important experimental evidence for the potential application of *Neobacillus* in plant disease management. Endophytic bacteria may inhibit the conidia development of *B*. *graminis* f. sp. *poae* and damage the pathogen cell structure; promote the robust growth of plant roots, indirectly enhancing their disease resistance; and stimulate the host’s immune system to strengthen the plant’s resistance to powdery mildew. The future research should focus on characterizing the metabolites of isolate 718 and the associated metabolic changes in the host during pathogen challenge to define the mechanistic foundation of this biocontrol interaction.

The management of powdery mildew can be effectively approached through the principles of integrated pest management (IPM). Biological control using endophytes can be integrated with chemical methods and the deployment of resistant plant varieties. However, since chemical agents may suppress beneficial endophytes, simultaneous application should be avoided. Although the effectiveness of endophytes in resistant varieties is somewhat reduced compared to susceptible ones, they still contribute to enhanced resistance, which remains superior to that of susceptible varieties. In summary, a coordinated combination of biological, chemical, and genetic strategies offers a sustainable path toward effective powdery mildew control.

### 3.3. Endophytic Bacterial Community Dynamics and Powdery Mildew Resistance in Kentucky Bluegrass

Numerous studies have indicated that phyllosphere microorganisms contribute to plant defense against pathogens [45]. In this study, high-throughput sequencing was employed to analyze the composition and dynamics of endophytic bacterial communities in Kentucky bluegrass. The results revealed that the richness and diversity of endophytic bacteria in leaf tissues were higher in healthy plants than in those inoculated with powdery mildew. This trend persisted after the application of endophytic bacteria, consistent with the findings of Sai Guo et al., who reported higher microbial diversity in healthy *Salvia miltiorrhiza* compared to susceptible plants [46]. These results suggest a strong association between disease resistance and microbial diversity in Kentucky bluegrass, though the underlying mechanisms require further investigation. At the phylum level, the endophytic bacterial community in Kentucky bluegrass leaves was predominantly composed of Proteobacteria, Firmicutes, Bacteroidetes, Actinobacteria, Acidobacteria, Gemmatimonadetes, Desulfobacteria, Myxococcota, and Nitrospirota. Treatment with isolate 718 increased the relative abundance of Proteobacteria in the endophytic bacterial community, suggesting a potential role of this phylum in enhancing host resistance to powdery mildew. For instance, members of *Pseudomonas* and *Acinetobacter* in Proteobacteria are known to inhibit pathogen growth through the production of antimicrobial metabolites. *Acinetobacter* sp. strain CRV19 as a biocontrol agent enhanced the resistance of grapevine against *Botrytis cinerea*. by up-regulation of the genes *PR-1*, *PR-5*, β-1,3-glucanase, and class III chitinase [47]. *Pseudomonas* sp., endophytic bacteria isolated from the roots of *Artemisia* sp., can induce resistance against pathogens such as *Verticillium dahliae*, *Colletotrichum gloeosporioides*, *Fusarium oxysporum*, and *Phytophthora capsici* [48]. The *Pseudomonas* genus is rich in species with the potential for biocontrol with positive effects on plant welfare, which actively participate in complex plant-pathogen-antagonist interaction. The most common molecules involved in this mechanism are 2,4-diacetylphloroglucinol, phenazine-1-carboxylic acid, phenazine-1-carboxamide, pyoluteorin and pyrrolnitrin. *Pseudomonas* spp. produce chitinases, glucanases and proteases involved in the suppression of many fungal diseases. Their production is mainly induced by the presence of fungal pathogen biomass and their cell wall [49]. It is indicated that Proteobacteria in the phyllosphere of Kentucky bluegrass after the application of endophytic bacteria may enhance the resistance of Kentucky bluegrass to powdery mildew by producing antibacterial substances or inducing systemic resistance of Kentucky bluegrass. At the same time, this study found that the dominant endophytic bacterial communities of Kentucky bluegrass inoculated with endophytic bacteria and inoculated with powdery mildew were Burkholderiales, Burkholderiaceae and *Cupriavidus*, while *Cupriavidus* were affiliated to Proteobacteria, Burkholderiales, Burkholderiaceae. Liu et al. studied the mechanism of organic compounds produced by *Burkholderia pyrrocinia* strain JK−SH007 induced resistance to poplar fusarium wilt at the molecular level [20], indicating that Kentucky bluegrass may enhance resistance to powdery mildew through organic substances produced by Burkholderiaceae after application of endophytic bacteria. In summary, the application of isolate 718 promoted the increase in some specific beneficial bacteria in Kentucky bluegrass in response to powdery mildew infection. Future work will elucidate the influence of isolate 718 on both endophytic and epiphytic fungal communities in Kentucky bluegrass and evaluate the stability of its powdery mildew control efficacy under field conditions.

## 4. Materials and Methods

### 4.1. Plant and Pathogen Materials

Kentucky bluegrass cultivars ‘Taihang’, ‘Black Jack’ and ‘Award’ were used in this study. Among them, ‘Taihang’ exhibits moderate resistance to powdery mildew. ‘Black Jack’ is moderately susceptible to powdery mildew and was supplied by Beijing Clover Seed and Turf (Beijing, China). The highly susceptible cultivar ‘Award’ was provided by Beijing Rytway Seed (Beijing, China) [50].

*B. graminis* f.sp. *poae* strain BGP (TG) was isolated and purified from the diseased *Poa pratensis* L. in the greenhouse (37°25′ N, 112°35′ E)by single spore heap separation method [50,51]. BGP (TG) was maintained and preserved on the highly susceptible Kentucky bluegrass ‘Endurance’ provided by Beijing Clover Seed and Turf Co., Ltd. (Beijing, China).

### 4.2. Planting Management and Preservation of Pathogen BGP (TG)

Uniform in size and plump seeds of four Kentucky bluegrass cultivars were selected for experiments. Seeds of ‘Taihang’, ‘Endurance’, ‘Black Jack’, and ‘Award’ cultivars, which were uniform in size and plump, were selected for use. Surface disinfection was conducted by immersing the seeds in 75% ethanol for 1 min, after which they were rinsed 3–5 times with sterile water to remove residual ethanol. A seedling substrate was prepared by thoroughly blending a mixture of potting soil and sand in a 2:1 ratio. This substrate was then placed into 32-cell seedling trays (dimensions: 540 mm × 280 mm × 110 mm) and leveled. Kentucky bluegrass seeds were sown evenly at a density of 10 g/m^2^ and covered with a 2–3 mm layer of vermiculite [51].

After sowing, the trays were transferred to an artificial climate incubator (Shanghai Boxun Medical Biological Instrument, Shanghai, China). The conditions were set as follows: a 16 h/8 h light/dark cycle, a constant temperature of 20 °C, and relative humidity maintained at 60 ± 2%. Irrigation was performed every five days. *Poa pratensis* L. plants, after a 30−day growth period, were inoculated via a dense conidial shake method. The inoculum source was powdery mildew conidia collected from leaves that had shown disease symptoms for 10 days, applied at a conidia density of 100–200 per mm^2^ [52].

### 4.3. Isolation and Identification of Endophytic Bacteria

#### 4.3.1. Isolation and Purification of Endophytic Bacteria

Luria–Bertani (LB) medium was prepared with the following composition: 10.0 g/L sodium chloride, 10.0 g/L tryptone (Beijing Solarbio Science Technology, Beijing, China), 5.0 g/L yeast extract (Beijing Solarbio Science Technology, Beijing, China), and 18.0 g/L agar (for solid medium), dissolved in 1000 mL of distilled water. The pH was adjusted to 7.0–7.5. For liquid medium, agar was omitted [53,54].

Leaf and root samples of the resistant Kentucky bluegrass ‘Taihang’ were collected and rinsed with sterile water in an ultra−clean bench (Shanghai Boxun Medical Biological Instrument, Shanghai, China). The plant materials were surface-disinfected by sequential immersion in 5% sodium hypochlorite for 3 min and 75% ethanol for 1 min, followed by rinsing with sterile water 5–7 times to remove residual disinfectants. The final rinse water was collected and used as a control.

After washing, 0.2 g of ‘Taihang’ leaf and root samples were added to a centrifuge tube with 1 mL of sterile water and homogenized using a tissue grinder (Shanghai Jingxin Industrial Development, Shanghai, China). The homogenate was serially diluted to generate four concentration gradients: 10^−1^, 10^−2^, 10^−3^, and 10^−4^. Then, 5 μL of each dilution was spread onto LB solid medium using glass beads. Three biological replicates were prepared for each dilution. Simultaneously, the final rinse water was also plated onto a blank LB plate for cultivation and used to amplify the 16S rRNA gene conserved sequence of the bacteria. If no microbial growth was observed on the control plate and no amplification occurred, it confirmed that the surface disinfection was effective and that the isolated bacteria represented endophytic bacteria from Kentucky bluegrass.

The plates were inverted and incubated in the dark at 28 °C for 48 h. After colony formation, distinct isolates were selected based on phenotypic characteristics, then purified and assigned isolates numbers. Following five successive rounds of purification, single colonies were picked and preserved on LB solid medium at 4 °C, as well as in 30% glycerol stock stored at −80 °C.

#### 4.3.2. Identification of Endophytic Bacteria

The isolated and purified endophytic bacteria of Kentucky bluegrass were placed in a sterile 96−well plate containing 50 μL sterile water per well. After vortex mixing, the bacterial suspensions were heated at 95 °C for 7 min in a PCR instrument to serve as DNA template for PCR amplification. Amplification of the bacterial 16S rRNA gene was performed using the universal primers 27F and 1492R [55]. The PCR reaction mixture (50 μL) consisted of: 25 μL of 2× Taq Master Mix (Vazyme Biotech, Nanjing, China), 2 μL of the 10 μM forward primer, 2 μL of the 10 μM reverse primer, 2 μL of 20 ng/μL DNA template, and 19 μL of ddH_2_O. The amplification protocol included initial denaturation at 94 °C for 3 min; followed by 30 cycles of denaturation at 94 °C for 30 s, annealing at 55 °C for 30 s, and extension at 72 °C for 1 min; with a final extension at 72 °C for 5 min. The resulting PCR products were sent to Sangon Biotech (Shanghai, China) for sequencing. For each PCR product, bidirectional sequencing was performed using the forward primer (27F) and the reverse primer (1492R). For each sample, at least three independent PCR amplifications were sequenced to confirm reproducibility. The obtained 16S rRNA gene sequences were assembled, annotated via BLASTN on the NCBI database and submitted to GenBank to obtain accession numbers (PX352499–PX352516). The MEGA11 software [56] was used to analyze the phylogenetic relationship between the sequence and the similar sequence in the system, and the neighbor-joining method was used and the bootstrap value was 1000 times.

### 4.4. Inhibition of Conidia Germination and Control Efficacy of Endophytic Bacteria on Powdery Mildew

#### 4.4.1. Preparation Method of Endophytic Bacteria Suspension

The preserved endophytic bacterial isolates of Kentucky bluegrass were first streaked onto LB solid medium using a sterile inoculating loop and incubated at 28 °C for 20 h in the dark. A single colony was then transferred into LB liquid medium and cultured under dark conditions at 28 °C with shaking at 180 rpm for 20 h. Subsequently, 8 mL of the bacterial suspension was centrifuged at 10,000 rpm and 4 °C for 10 min. After discarding the supernatant, the cell pellet was collected and resuspended in sterile water to adjust the OD600 to the desired value for subsequent experiments.

#### 4.4.2. Conidia Germination Inhibition Experiment of Kentucky Bluegrass Powdery Mildew

The prepared endophytic bacterial suspension from Kentucky bluegrass was mixed with 2% melted agar in a 1: 1 ratio and applied to a slide to form a thin layer agar medium. A pesticide treatment group was also established, in which 200 mg/L of 20% triadimefon EC was blended with 2% melted agar at a 1:1 ratio to create a similar thin-layer medium. Using a dense shaking method, conidia of Kentucky bluegrass powdery mildew were dispersed onto the medium. Each treatment included three biological replicates. The conidia germination rate on the thin layer agar medium mixed with sterile water and 2% melted agar was used as the control. After an 18 h incubation period at 20 °C in the dark, the inhibition rate of conidia germination was assessed using a light microscope (Novel, Ningbo, China) [14,57,58]. For each treatment, 50 conidia were examined to determine their germination status. The inhibition rate of conidia germination was calculated according to the following formula:

The inhibition rate of conidia germination (%) = [Control conidia germination rate − Treatment conidia germination rate)/Control conidia germination rate] × 100 [57].

#### 4.4.3. Determination Method of Control Efficacy of Different Endophytic Bacteria on Powdery Mildew

When Kentucky bluegrass reached 30 days of growth, powdery mildew was inoculated using a dense conidial shaking method. Different endophytic bacterial suspensions (10^9^ CFU/mL) were sprayed at 24 h before and 24 h after powdery mildew inoculation. A 200 mg/L solution of 20% triadimefon EC was used as a chemical control, and water was applied as a blank control. Each treatment applied 5 mL of bacterial suspension, with three biological replicates per treatment. For each replicate, 25 seedlings were randomly selected for assessment. After inoculation of powdery mildew, the disease severity was counted and the disease index (DI) and control efficacy were calculated after 10 days of culture at 20 °C in the artificial climate incubator (Shanghai Boxun Medical Biological Instrument, Shanghai, China).

The severity of disease on leaves was assessed, and the disease index along with control efficacy were calculated according to the following grading criteria for Kentucky bluegrass powdery mildew: 0: Asymptomatic; 1: sparse and faint powdery mildew present, with infected area covering less than 5% of the total leaf area; 3: thin white powdery layer visible, covering 6–10% of the total leaf area; grade 5: thick white powdery layer, covering 11–20% of the total leaf area; grade 7: thick white powdery layer, covering 21–40% of the total leaf area; grade 9: thick white powdery layer, covering more than 40% of the total leaf area [9].

DI = [(∑number of diseased leaves at all levels × relative level value)/(total number of leaves surveyed × 9)] × 100

Control efficacy (%) = [(Control disease index − Treatment disease index)/Control disease index] × 100

#### 4.4.4. Control Efficacy of Different Concentrations of Endophytic Bacteria on Powdery Mildew

The control efficacy of the endophytic bacterial suspension against powdery mildew was evaluated at three concentrations: 10^7^, 10^9^, and 10^11^ CFU/mL.

### 4.5. Sample Collection for Microbiome Analysis

Collected leaf samples were surface-disinfected (as described in Section 4.3.1), immediately frozen in liquid nitrogen, and stored at −80 °C until further analysis. The experiment included four treatments: (1) MM: application of water without inoculation of powdery mildew; (2) MB: application of water with inoculation of powdery mildew; (3) BM: application of isolate 718 bacterial suspension without inoculation of powdery mildew; (4) BB: application of isolate 718 bacterial suspension with inoculation of powdery mildew. Each treatment consisted of five biological replicates, with one gram of Kentucky bluegrass leaves collected from each replicate for analysis.

### 4.6. High−Throughput 16S Ribosomal RNA Gene Sequencing

Total genomic DNA was extracted from the samples using the TGuide S96 Magnetic Bead DNA Kit (Tiangen Biotech, Beijing, China), following the manufacturer’s instructions. The hypervariable V3-V4 region of the bacterial 16S rRNA gene was amplified by polymerase chain reaction (PCR) with the primer pair 338F and 806R [59]. The resulting PCR products were purified, and paired-end sequencing (2 × 250 bp) was conducted on the Illumina NovaSeq 6000 platform. Paired-end reads were filtered to eliminate the low-quality reads (with a quality score lower than 20) via quality filter using Trimmomatic 0.33 with rigorous filtering criteria. The sequential noise reduction method (dada2) was performed to optimize the data processing of the control quality.

### 4.7. Bioinformatic Analysis

Qualified sequences were clustered into OTUs at a 97% similarity threshold using USEARCH (version 10.0). OTUs were filtered with a threshold set at 0.005% of the total number of sequences [60]. Taxonomic annotation was performed based on the Silva138 reference database, from which species classification information for each sequence was obtained. The community composition of endophytic bacteria in each Kentucky bluegrass sample was profiled at multiple taxonomic levels (kingdom, phylum, class, order, family, genus and species). Alpha diversity was calculated and displayed by the QIIME2 (version 2024.10) and R software (version 4.4.0), respectively, by calculating library coverage, Shannon, Simpson, ACE, and Chao1 indices. The rarefaction curve and rank abundance curve, the Venn diagram illustrating bacterial community OTUs, the relative abundance of the top 10 bacterial phyla and genera across different treatments of *Poa pratensis* L. were displayed by R software. Additionally, principal coordinate analysis (PCoA) was performed based on Bray–Curtis distances computed in QIIME2. Linear Discriminant Analysis (LDA) effect size (LEfSe) was employed to test the significant taxonomic difference among group. A logarithmic LDA score of 4.0 was set as the threshold for discriminative features.

### 4.8. Analysis of Data

Data processing and statistical analyses were performed using Microsoft Excel 2019 and IBM SPSS Statistics (version 26.0). Differences among groups were assessed by one-way or two-way analysis of variance (ANOVA) followed by Tukey honest significant difference (HSD) test. Figures were generated using Origin 2021.

## 5. Conclusions

In this study, 18 endophytic bacteria were isolated from the leaf and root samples of the resistant variety Kentucky bluegrass ‘Taihang’, including 3 isolates of Proteobacteria, 6 isolates of Actinobacteria and 9 isolates of Firmicutes. The conidia germination inhibition test showed that the inhibition effect of isolates 6213 and 718 on the conidia germination of *B. graminis* f. sp. *poae* was better than that of 16 other endophytic bacteria, and the germination inhibition rate was as high as more than 80%. Therefore, they were selected to determine the incidence and control effect of powdery mildew in different resistant varieties of *P*. *pratensis*. The results showed that the control effect of isolate 718 was better than that of isolate 6213. By further determining the incidence and control effect of isolate 718 with different concentrations on powdery mildew of different resistant varieties of Kentucky bluegrass, the results showed that the control effect of isolate 718 with 10^9^ CFU/mL on powdery mildew of Kentucky bluegrass was the best, which could be used as a potential biological control agent and applied. The abundance of Proteobacteria on Kentucky bluegrass after the application of isolate 718 may enhance the resistance of Kentucky bluegrass to powdery mildew, and the dominant endophytic bacterial communities were Burkholderiales, Burkholderiaceae and *Cupriavidus*, indicating that *Poa pratensis* L. responded to powdery mildew infection under the regulation of isolate 718. However, these findings are based on controlled conditions, and further field validation is required to assess the ecological stability and practical efficacy of isolate 718. Future work should also focus on elucidating the underlying mechanisms—such as metabolite production or signaling pathways—through which this isolate influences both the microbiome and plant immunity. This study highlights the promise of microbiome-mediated resistance and lays a foundation for developing sustainable IPM strategies against powdery mildew.

## Figures and Tables

**Table 1:** The information of 16S rRNA gene sequence of endophytic bacteria isolated from *Poa pratensis* L.

The Name of Isolates	Length of 16S rRNA Gene Sequence (bp)	Accession Number of Isolates	Closest Relative Species	Accession Number of Reference Stains	Similarity (%)
6211	1425	PX352511	*Arthrobacter globiformis*	NR_112192.1	98.80
6212	1453	PX352512	*Paenibacillus xylanexedens*	NR_044524.1	98.96
6213	1450	PX352513	*Bacillus arachidis*	NR_041248.1	99.77
6214	1428	PX352514	*Streptomyces ossamyceticus*	NR_118277.1	99.85
6215	1419	PX352515	*Curtobacterium flaccumfaciens*	NR_025467.1	99.58
6217	1423	PX352516	*Kocuria rosea*	NR_044871.1	99.51
711	1447	PX352499	*Lysobacter prati*	NR_180692.1	99.00
712	1452	PX352500	*Paenibacillus illinoisensis*	NR_115624.1	99.50
713	1452	PX352501	*Fredinandcohnia onubensis*	NR_149252.1	99.02
714	1378	PX352502	*Pseudarthrobacter siccitolerans*	NR_108849.1	99.56
715	1446	PX352503	*Cytobacillus firmus*	NR_025842.1	98.75
716	1451	PX352504	*Cytobacillus firmus*	NR_025842.1	98.75
717	1435	PX352505	*Variovorax soli*	NR_114300.1	99.16
718	1450	PX352506	*Neobacillus drentensis*	NR_114085.1	98.56
719	1457	PX352507	*Metabacillus bambusae*	NR_180924.1	99.16
720	1459	PX352508	*Paenibacillus silvae*	NR_156836.1	99.02
721	1421	PX352509	*Pseudarthrobacter psychrotolerans*	NR_174315.1	99.20
722	1441	PX352510	*Acinetobacter calcoaceticus*	NR_114922.1	99.79

## Data Availability

Data is contained within the article.
